# Spatial distribution of bacterial communities driven by multiple environmental factors in a beach wetland of the largest freshwater lake in China

**DOI:** 10.3389/fmicb.2015.00129

**Published:** 2015-02-26

**Authors:** Xia Ding, Xiao-Jue Peng, Bin-Song Jin, Ming Xiao, Jia-Kuan Chen, Bo Li, Chang-Ming Fang, Ming Nie

**Affiliations:** ^1^School of Life Sciences and Institute of Life Science, Nanchang UniversityNanchang, China; ^2^School of Environmental and Biological Engineering, Nanjing University of Science and TechnologyNanjing, China; ^3^College of Life and Environment Science, Shanghai Normal UniversityShanghai, China; ^4^Ministry of Education Key Lab for Biodiversity Science and Ecological Engineering, The Institute of Biodiversity Science, Fudan UniversityShanghai, China

**Keywords:** bacteria, community structure, freshwater lake wetland, Poyang Lake, spatial distribution, 16S rRNA

## Abstract

The spatial distributions of bacterial communities may be driven by multiple environmental factors. Thus, understanding the relationships between bacterial distribution and environmental factors is critical for understanding wetland stability and the functioning of freshwater lakes. However, little research on the bacterial communities in deep sediment layers exists. In this study, thirty clone libraries of 16S rRNA were constructed from a beach wetland of the Poyang Lake along both horizontal (distance to the water-land junction) and vertical (sediment depth) gradients to assess the effects of sediment properties on bacterial community structure and diversity. Our results showed that bacterial diversity increased along the horizontal gradient and decreased along the vertical gradient. The heterogeneous sediment properties along gradients substantially affected the dominant bacterial groups at the phylum and species levels. For example, the NH^+^_4_ concentration decreased with increasing depth, which was positively correlated with the relative abundance of *Alphaproteobacteria.* The changes in bacterial diversity and dominant bacterial groups showed that the top layer had a different bacterial community structure than the deeper layers. Principal component analysis revealed that both gradients, not each gradient independently, contributed to the shift in the bacterial community structure. A multiple linear regression model explained the changes in bacterial diversity and richness along the depth and distance gradients. Overall, our results suggest that spatial gradients associated with sediment properties shaped the bacterial communities in the Poyang Lake beach wetland.

## Introduction

Wetland ecosystems are considered the most biologically diverse ecosystems (Iasur-Kruh et al., 2009; Wang et al., 2012). A beach wetland is a landform along the edge of a body of water and is an interface between the land and water. Bacteria are ubiquitous and play key roles in ecosystem functioning, including cycling of the majority of biologically active elements (Woese, 1990; Woese et al., 1990; Gucht et al., 2007; Newton et al., 2011). However, systematic exploration of geographic bacterial patterns through the simultaneous consideration of contemporary environmental variations and stereoscopic spatial distribution (distance and depth) is largely lacking, resulting in a poor understanding of how environmental factors shape bacterial communities in beach wetlands of lake ecosystems (Yannarell and Triplett, 2005; Córdova-Kreylos et al., 2006; Zhou et al., 2008).

Recent studies demonstrate that bacterial communities in lake wetland ecosystems are strongly correlated with a multitude of environmental factors over horizontal gradients ranging from hundreds of kilometers to centimeters (Terrados et al., 1999; Yannarell and Triplett, 2004; Crump et al., 2007). Several studies suggest that bacterial distributions may be spatially predictable rather than random (Ettema and Wardle, 2002). Differences in environmental factors along sediment horizontal gradients largely determine bacterial composition and diversity, such as water content (Drenovsky et al., 2004; Badin et al., 2011), C and N availability (Cookson et al., 2008; Moseman-Valtierra et al., 2010; Mackelprang et al., 2011; Lin et al., 2012), temperature (Hall et al., 2008; Redmond and Valentine, 2011), pH (Lindström et al., 2005), and sediment structure characteristics (Liu et al., 2011). Despite the importance of sediment bacteria in biogeochemical cycling, the bacterial communities in the deeper layers are not well studied (Haglund et al., 2003; Luna et al., 2004); the bacterial communities of the sediment surface layers have been far better studied than those of the deeper layers (Liao et al., 2009; Schauer et al., 2010). Expanding our knowledge of bacterial diversity and distribution from the surface to deeper sediment layers will improve our understanding of biodiversity and functioning of beach sediment.

**Figure 1:**
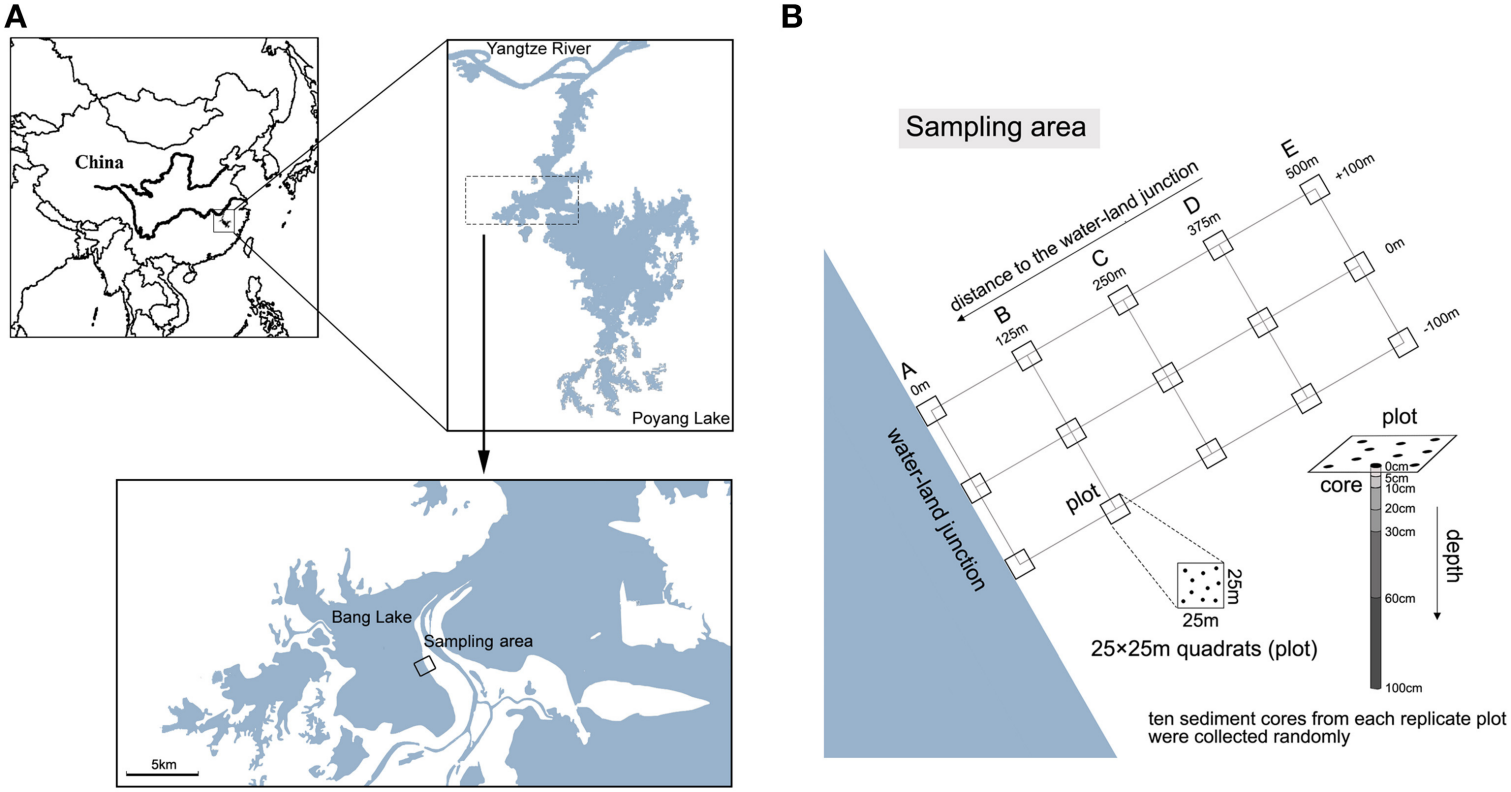
**Maps of sampling stations. (A)** The sampling sites in the Bang Lake of the Poyang Lake wetland. **(B)** Core sediment samples were collected from a range of beach wetland locations along gradients of depth and distance to the water-land junction. We sampled 5 sediment locations (A, B, C, D and E), which were 0, 125, 250, 375, and 500 m from the water-land junction, respectively. Three 25 × 25 m quadrats (plots) that were 100 m apart were established in each location. From each replicate plot, 10 sediment cores (diameter 2.5 cm) were collected and pooled.

In this study, we evaluated the spatial distribution of bacterial communities along gradients of both sediment depth and distance to the water-land junction in the Poyang Lake wetland (Figure 1) (Jiangxi Province, mid-China), the largest freshwater lake in China. The aim of this study was to determine whether main bacterial communities are regularly distributed along vertical and horizontal gradients and what environmental factors affect the spatial distributions of bacterial communities. The spatial distribution of the bacterial communities was determined by constructing clone libraries of 16S rRNA and analyzing the associations between the different communities. We hypothesized that different geochemical parameters along vertical and horizontal gradients affect specific bacterial groups in beach sediments.

## Methods

### Study location and sampling

Poyang Lake is located in the northern part of the Jiangxi Province and at the southern bank of the middle reaches of the Yangtze River (29°07′N, 115°59′E) (Figure 1A) (Liu et al., 2011). The lake covers 3283 km^2^ during the rainy season. The mean annual temperature and precipitation are 17°C and 1636 mm, respectively. The Poyang Lake wetland has a high fluctuation of seasonal water levels between the flood and dry seasons (Xu et al., 2014). In the dry season, the surface area of the lake shrinks to less than one-tenth of the area in the flood season. We sampled beach sediments between the dry and flood seasons, because this stage could capture the major characteristics of beach wetland and obtain great differences in geochemical parameters (e.g., water content) along the spatial gradients. In July 2011, sediment samples were collected from a range of beach wetland locations along gradients of depth and distance to the water-land junction on the shore of Bang Lake, which is an isolated sub-lake located in the core area of Poyang Lake. We chose this lake for avoiding disturbances from agricultural and grazing activities. We sampled 5 sediment locations (A, B, C, D and E), located 0, 125, 250, 375, and 500 m from the water-land junction, respectively. Three 25 × 25 m quadrats (plots) that were 100 m apart were established in each location. From each replicate plot, 10 sediment cores were collected using side opening steel tubes (length 1.2 m, diameter 2.5 cm). Next, the samples were pooled and sieved (mesh size < 2 mm) to remove stones and plant materials. At each location, we sampled 6 depth strata per transect: 0–5, 5–10, 10–20, 20–30, 30–60, and 60–100 cm (Figure 1B). Samples for sediment chemical analysis were stored at 4°C, and samples for clone library analysis were stored at −20°C. All samples were processed within 4 h of collection. Detailed descriptions of the sampling coordinates, sediment chemical properties, sediment depth, and distance to the water-land junction are listed in Table S1.

### Sediment physicochemical analyses

To understand physiochemical effects from the spatial distances, water content (WC), sediment bulk density (SBD), sediment organic carbon (SOC), and inorganic nitrogen (NH^+^_4_, NO^−^_3_) were determined. These sediment physicochemical variables were shown to have low co-correlations with each other because the average correlation coefficient *r* value is 0.27 and only 4 of 15 correlations between these variables are significant (*P* < 0.05) (Table S2). WC was determined by weighing a sediment sample before and after drying at 105°C for 24–48 h to a constant weight. SBD was determined by oven-drying sediment cores of a fixed volume (Cui et al., 2012). Sediment pH was measured on sediment slurry at a 2.5:1 water: sediment ratio using a glass electrode (Meng et al., 2012). SOC was measured with a TOC analyzer (Analytikjena HT1300, Germany) after removing sediment carbonates using 1 M HCl. Inorganic nitrogen (NH^+^_4_, NO^−^_3_) was extracted and measured using 2 M KCl and a discrete auto analyzer (Smartchem 200, Westco, France).

### DNA extraction, clone library construction and sequencing

The total genomic DNA of sediment samples was extracted from 0.5 g (fresh weight) of the sediment sample with the Fast DNA Spin kit for sediments (Qbiogene, Irvine, CA) according to the manufacturer's instructions. After extraction, the DNA samples were immediately frozen at −80°C for further analysis.

The template DNA isolated from the subsamples of each location was pooled so that each subsample was equally represented. The pooled DNA (20 ng for each sample) was analyzed using PCR (predenaturation step of 5 min at 95°C followed by 30 cycles of 1 min at 94°C, 30 s at 53°C and 2 min at 72°C, followed by a final elongation step of 72°C for 15 min), with the bacteria-specific primers 27F (5′-AGA GTT TGA TCM TGG CTC AG-3′) and 1492R (5′-TAC GGY TAC CTT GTT ACG ACT T-3′) (Lane, 1991; Meng et al., 2012). The PCR products were purified using the QIAquick PCR Purification Kit (Qiagen, Germany) and quantified using Nano Drop ND-3000 (Nano-Drop Technologies). The PCR products were subsequently cloned into the pMD18-T vector system (TaKaRa, Japan) and transformed into *Escherichia coli* Top 10. A total of 2520 recombinant clones were individually chosen from the 30 clone libraries, and partial 16S rRNA gene sequences were determined using a BigDye Terminator V3.1 Cycle Sequencing Kit (Applied Biosystems, Foster City, CA) and an ABI3730 PRISM Genetic Analyzer (Applied Biosystems).

### Sequence processing and operational taxonomic unit (OTU) clustering

A total of 2380 sequences from 30 clone libraries (each clone library had 73 to 86 sequences) were retained and deposited into the GenBank database under the accession numbers (KJ013600–KJ015979).

The Ribosomal Database Project (RDP; http://rdp.cme.msu.edu/) classifier was used to assign 16S rRNA gene sequences (Maidak et al., 2001). The sequences were screened and sorted for chimeras within Mothur using the chimera.uchime command (http://www.mothur.org/wiki/Main_Page). The OTU clustering was performed by setting a 0.03 distance limit (equivalent to 97% similarity) using the Mothur program (Schloss et al., 2009).

### Statistical methods for community analyses

Canonical Correspondence Analysis (CCA) and Principal Component Analysis (PCA) were used to identify the most important abiotic factors to the bacterial community composition. This analysis was performed using a Multivariate Statistical Package (MVSP) (Kovach Computing, Anglesey, United Kingdom). The relationships between the relative abundance of bacterial species and the taxonomic diversity for groups with shared physico-chemical features were tested with linear regression analyses using SigmaStat 3.5/SigmaPlot 10.0 (SysStat Software Inc., CA).

## Results

### Sediment physico-chemical characteristics

A total of 30 samples were collected from 5 different locations along a distance gradient, and samples from each of the 6 different depths were collected at each location. The major geographical and physiochemical characteristics of the lake sediments are summarized in Table S1. Across the sampling sites, the WC varied from 32.2 to 23.2%, and the SOC varied from 25.09 to 6.03 g·kg^−1^. The WC and SOC were highly correlated with the geographic distance to the water-land junction, with both WC (*P* < 0.001) and SOC (*P* < 0.05) decreasing as the distance to the water-land junction increased. The SBD varied from 0.27 to 0.62 g·cm^−3^, and the NH^+^_4_ concentration ranged from 14.92 to 107.86 mg·kg^−1^. Both the SBD and NH^+^_4_ were significantly correlated with geographic depth (all *P* < 0.001). The SBD increased with increasing depth, and NH^+^_4_ concentration decreased with increasing depth (Table S2). No significant spatial differences were observed in the sediment pH, which varied from 6.4 to 7.9.

### Distribution of taxa and phylotypes

The clone libraries were built with careful consideration of sequence quality to ensure significant clone coverage. Across all sediment samples, we obtained a total of 2380 sequences, with 480–510 sequences per location (mean = 500) (Table S3). We successfully classified 79.7% of the obtained sequences.

**Figure 2:**
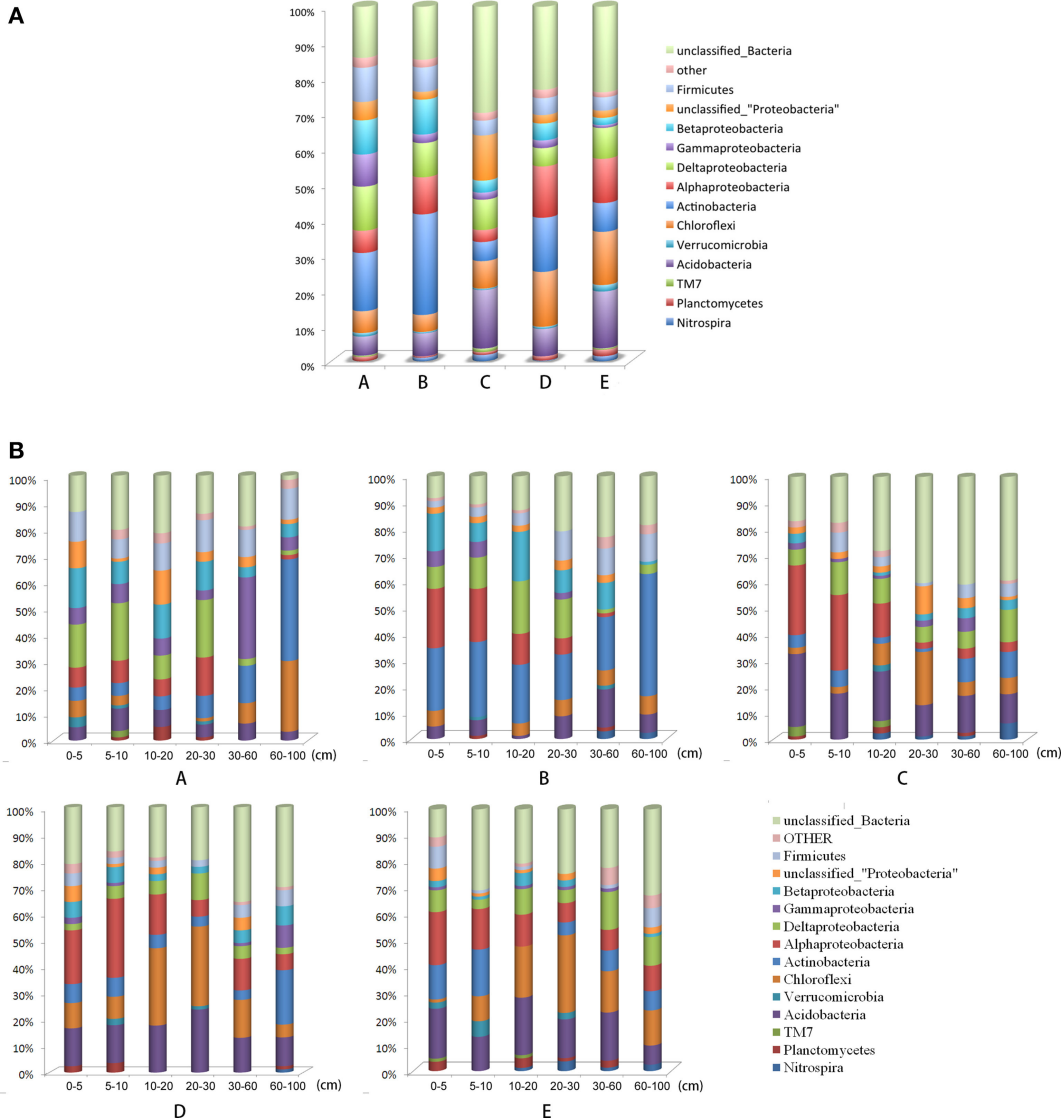
**Relative abundances of the dominant bacterial phyla in the sampling locations**. Relative abundances were calculated by the proportional frequencies of the DNA sequences that could be classified at the phylum level. **(A)** A, B, C, D, and E were five sediment locations, located 0, 125, 250, 375, and 500 m from the water-land junction, respectively. **(B)** At each location (A, B, C, D, and E), there were 6 depth strata sampled per transect: 0–5, 5–10, 10–20, 20–30, 30–60, and 60–100 cm.

The dominant phyla (relative abundance >5%) across all locations were *Proteobacteria* (32.61%), *Actinobacteria* (14.82%), *Acidobacteria* (10.38%), *Chloroflexi* (9.78%), and *Firmicutes* (5.89%). These phyla accounted for more than 73.48% of the collected bacterial sequences (Figure 2). *Spirochaetes*, *Nitrospira*, *Armatimonadetes*, *WS3*, *Chlorobi*, *Planctomycetes*, *Cyanobacteria/Chloroplast*, *Bacteroidetes*, *TM7*, *Verrucomicrobia*, and *Gemmatimonadetes* were present in most of the sediment samples with low relative abundance (<5%) (Figure 2, Table S4). The RDP database revealed the recovery of 21 phyla from lake epilimnia, with 5 of the phyla frequently commonly recovered (*Proteobacteria*, particularly *Betaproteobacteria*, with 4300 and 2600 sequences, respectively; *Actinobacteria* with 3000 sequences; *Bacteroidetes* with 1900 sequences; *Cyanobacteria* with 800 sequences; and *Verrucomicrobia* with 300 sequences) (Newton et al., 2011). However, both the phyla *Cyanobacteria* and *Bacteroidetes* were present in low relative abundance in Poyang Lake sediment.

The different taxonomic levels of the bacteria were shown to heterogeneously distribute along horizontal and vertical gradients. The decrease in bacterial relative abundance as distance from the water-land junctions increased was particularly sharp for *Firmicutes*, *Deltaproteobacteria*, and *Gammaproteobacteria* (Figure 2). However, the *Alphaproteobacteria*, *Chloroflexi*, and *Acidobacteria* relative abundance increased along the distance gradient (Figure 2). *Alphaproteobacteria* and *Deltaproteobacteria* were more abundant at depths less than 30 cm (Table S4).

**Figure 3:**
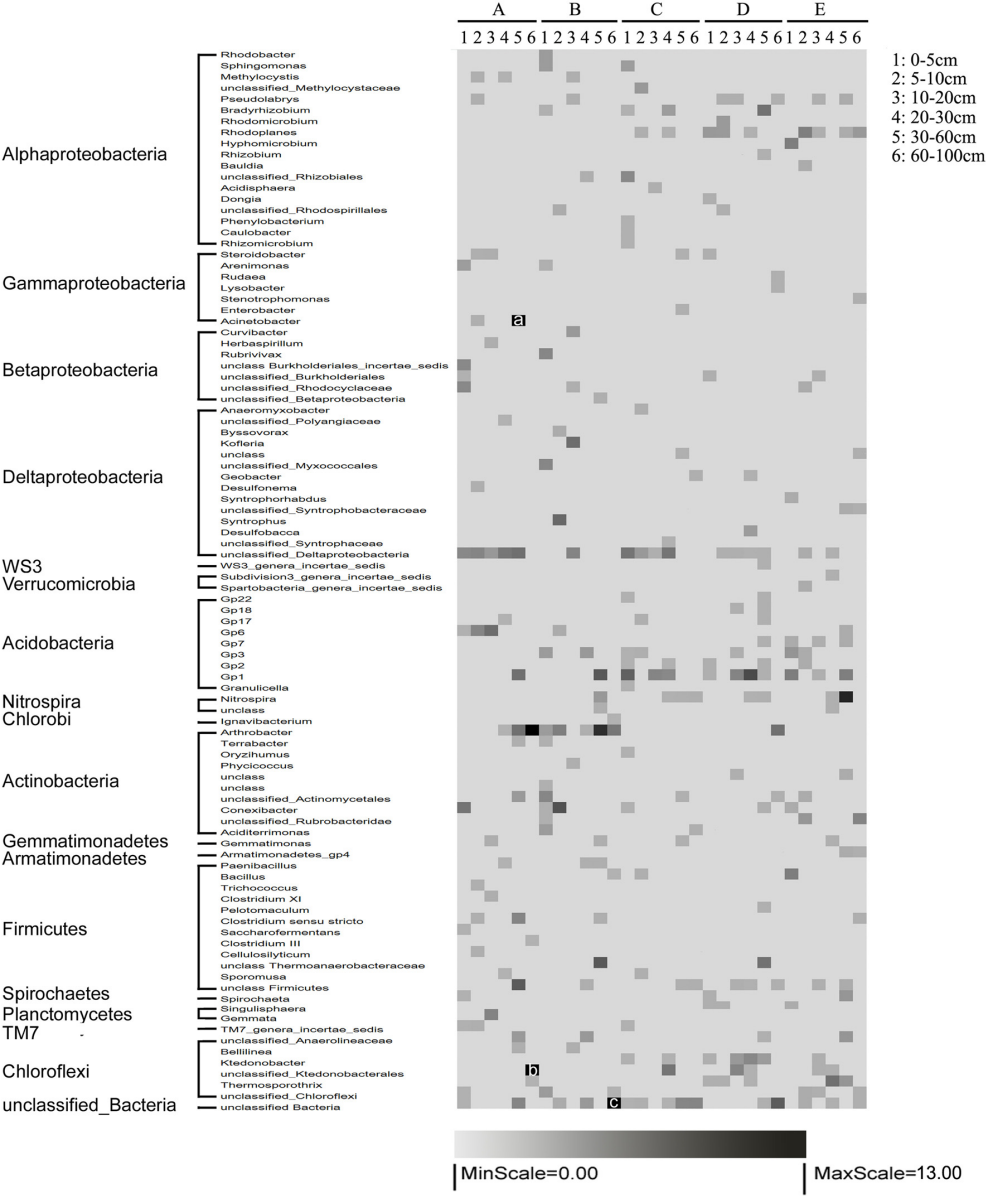
**Relative abundance distribution of the dominant operational taxonomic units (OTUs) (97% similarity)**. A, B, C, D, and E represent the five sediment locations, which were 0, 125, 250, 375, and 500 m from the water-land junction, respectively. Numbers 1, 2, 3, 4, 5, and 6 represent the six depth strata sampled per transect: 0–5, 5–10, 10–20, 20–30, 30–60, and 60–100 cm. The relative abundances of each OTU were normalized to have a mean of 0 and a standard deviation of 100%. The percent abundances of dominant genera in each location are indicated by shading: percentages close to 13 are indicated in black, and those close to 0 are in gray. The actual percentages of a, b, and c are 24, 14, and 19%, respectively.

The heterogeneous distribution along horizontal and vertical gradients was observed more clearly when the bacterial communities were classified at the genus level. *Acidobacteria*_Gp6, *Acidobacteria*_Gp2, *Acidobacteria*_Gp1, *Arthrobacter*, *Pseudolabrys*, *Thermosporothrix*, and *Ktedonobacter* were detected in nearly all the samples (Figure 3). Othergenera were found in a portion of the samples. Specifically, *Acidobacteria*_Gp3, *TM7*_genera_incertae_sedis and *Singulisphaera* were mainly distributed in surface sediments, whereas *Nitrospira* was mainly distributed in submerged sediments. *Methylocystis*, *TM7*_genera_incertae_sedis, and *Acinetobacter* were abundant in the water-land junctions, whereas *Rhodoplanes*, *Acidobacteria*_Gp7, and *Armatimonadetes*_gp4 were mainly distributed far from the water-land junctions (Figure 3).

### Relationship between bacterial community structure and sediment variables

**Figure 4:**
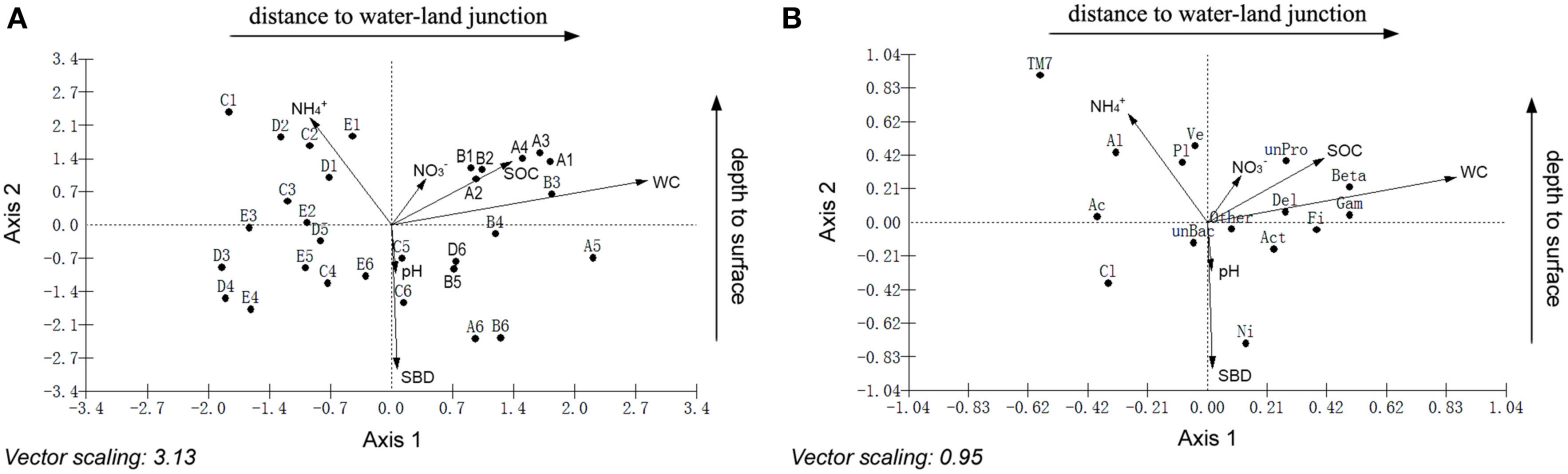
**Canonical correspondence analysis (CCA) showing the relationship between the sampling locations and sediment variables **(A)**, and the relationship between the relative abundances of the dominant bacterial phyla and sediment variables **(B)****. The direction of an arrow indicates the steepest increase in the variable, and the length indicates the strength relative to the other variables. The filled circle in **(A)** represents the sampling locations. A, B, C, D, and E were five sediment locations, which were 0, 125, 250, 375, and 500 m from the water-land junction, respectively. At each location, there were six depth strata sampled per transect: 0–5, 5–10, 10–20, 20–30, 30–60, and 60–100 cm, which were indicated by numbers 1, 2, 3, 4, 5, and 6, respectively. The filled circle in **(B)** represents the diversity of the bacteria. Bacterial group abbreviations are Pro, *Proteobacteria*; Al, *Alphaproteobacteria*; Beta, *Betaproteobacteria*; Gama, *Gammaproteobacteria*; Del, *Deltaproteobacteria*; Act, *Actinobacteria*; Ac, *Acidobacteria*; Cl, *Chloroflexi*; Fi, *Firmicutes*; Ni, *Nitrospira*; Pl, *Planctomycetes*; TM7; Ve, *Verrucomicrobia*. WC, water content; SBD, sediment bulk density; SOC, soil organic carbon.

Canonical correspondence analyses (CCA) were performed to examine the relationship between bacterial community structure and geochemistry. The results indicated that sediment chemical properties and geographical characteristics have different effects on sediment bacteria (Figure 4, Tables S5–S7). Axis 1 and axis 2 were interpreted as the distance to the water-land junction gradient and depth, respectively. The small angles between the WC, SOC and NO^−^_3_ vectors along the distance to the water-land junction indicated strong correlations among these variables. Similarly, the small angles between the pH, SBD and NH^+^_4_ vectors along the depth indicated strong correlations between these variables. The WC, SOC, and NO^−^_3_ showed strong positive correlations with axis 1 and positive correlations with axis 2. The SBD and pH showed strong negative correlations with axis 2 and positive correlations with axis 1. NH^+^_4_ showed a strong negative correlation with both the first and second axes. Among the sediment chemical properties, the WC, SBD, NH^+^_4_, and SOC were the most important factors in determining the bacterial community structure (Figure 4A). In addition, *Planctomycetes*, *Alphaproteobacteria*, *Verrucomicrobia*, and *Nitrospira* were significantly associated with NH^+^_4_, SBD, and depth. Furthermore, *Betaproteobacteria*, *Gammaproteobacteria*, *Acidobacteria*, and *Firmicute* were highly associated with the WC, SOC, and distance to the water-land junction (Figure 4B).

**Figure 5:**
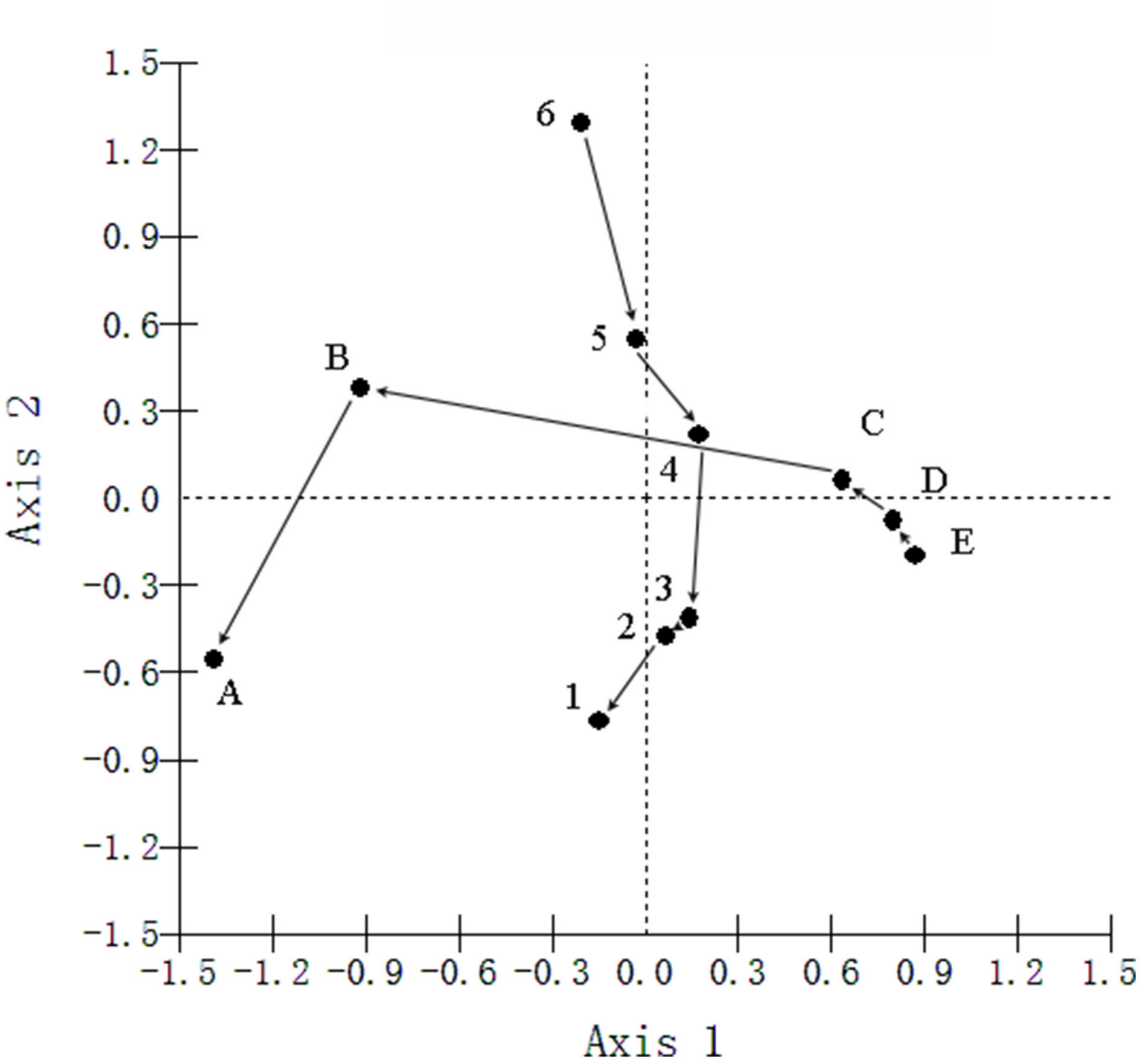
**Principal Component Analyses (PCA) of the bacterial communities, with symbols coded by depths or sites**. A, B, C, D, and E were the five sediment locations, which were 0, 125, 250, 375, and 500 m from the water-land junction, respectively. At each location, there were six depth strata sampled per transect: 0–5, 5–10, 10–20, 20–30, 30–60, and 60–100 cm, which were indicated by numbers 1, 2, 3, 4, 5, and 6, respectively.

We also preformed linear regression analyses to elucidate the relationships between bacterial relative abundances and sediment variables. The relative abundances of dominant bacterial phyla (*Proteobacteria*, *Alphaproteobacteria*, *Nitrospira, Betaproteobacteria*, *Acidobacteria*, and *Firmicute*) were significantly correlated with the sampling depth and the distance to the water-land junction (all *P* < 0.005) (Figure S1, Table S2). The sediment chemical properties (WC, SBD, and NH^+^_4_) were also significantly correlated with the distributions of these bacteria across sediment depth and sediment distance to the water-land junction (Figure S2, Table S2). These results were consistent with our above-mentioned CCA observations: sediment chemical properties were divided into two different “factors” determined by sediment depth and distance to the water-land junction (Table S2, Figures 3, 5).

### Changes in bacterial diversity along the depth and distance

#### Gradients

Furthermore, PCA was performed to test whether there was a difference between the depth and distance by clustering the samples according to depth regardless of site and clustering the samples according to site (regardless of depth) (Figure 5 and Table S8). The PCA biplot clearly revealed that the bacterial communities were shaped by both depth and distance. It was notable that Axis 1 changed more dramatically within the sites of A, B and C than in the sites of C, D and E (Figure 5).

## Discussion

Sediment characteristics of the Poyang Lake varied in space. Our samples were collected between the dry and flood seasons and therefore do not take into account the seasonal variability of the system. Nevertheless, although the results only provide a “snap-shot” of how spatial gradients shaped the bacterial communities, they suggest that multiple environmental factors along spatial gradients can strongly mediate beach bacterial communities in a subtropical freshwater wetland region of China.

The clone library analysis used here was able to target the dominant bacteria. However, the averaged 80 clones per sample could lead to miss some groups of important bacteria, resulting in underestimation of bacterial diversity. Our study suggests that *Proteobacteria*, *Acidobacteria*, and *Actinobacteria* were dominant prokaryotes. These bacteria have been documented as numerically important components in a geographically wide range of freshwater lake habitats, including lakes in North America (Newton et al., 2011), Europe (Glockner et al., 2000), Africa (Humbert et al., 2009), Asia (Wu et al., 2006), and Antarctica (Pearce et al., 2003). But our results suggest that bacterial community composition and diversity were driven by sediment properties (e.g., the WC and SOC), which differed over the depth and distance to the water-land junction spatial gradients. By analyzing the spatial distribution of bacterial communities across a small spatial scale (500 m), our results also indicated that sediment chemical properties were divided into two different “factors.” Spatial gradients in association with varying sediment properties drove bacterial community composition. In addition, our results suggest that different geochemical parameters along vertical and horizontal gradients can affect specific bacterial groups in sediments.

In this study, the WC and nutrient availability (e.g., SOC) significantly increased with increasing distance to the water-land junction. Sediment moisture along the distance to water-land junction exerted a selective pressure on the bacterial community. Sediments with a high of WC harbor the less *Acidobacteria* and *Chloroflexi*, and more *Betaproteobacteria* and *Firmicute*across locations (Figure 4, Figure S2). Nutrient availability along distance gradient is another important factor that influences the bacterial community (Logue and Lindström, 2008). The relative abundance of *Actinobacteria* decreased as increased SOC (Figure 4). Increased nutrient concentrations could select against the freshwater lake *Actinobacteria* (Haukka et al., 2006). Organic C in sediment primarily originates from living organisms, such as phytoplankton, plant tissue, and fish (Donohue and Garcia Molinos, 2009). In addition, our results indicated that SOC showed correlations with the abundance of *Betaproteobacteria* (Figure 4). Several studies also suggested that *Betaproteobacteria* growth is closely associated with the sediment nutrients (Chen and Chiu, 2000; Lin et al., 2010). The freshwater lake *Betaproteobacteria* is fast growing and nutrient loving (Newton et al., 2011), which was highly associated with SOC (Figure 4, Figure S1).

The majority of the *Alphaproteobacteria* belonged to *Rhizobiales*, which were more abundant in the surface layer sediment samples with low SBD (Figure 3). The SBD varied as the water content of the sediment changed due to deposition and subsequent compaction. SBD generally increases with depth and time as pore water is expelled from the sediment and transported to the surface (Boroujeni et al., 2009). In addition, the *Rhizobiales* are controller at the hub of the ecosystem N cycle, and often facilitate atmospheric N fixation by plants (Im et al., 2006; Yarwood et al., 2009). NH^+^_4_ is mainly produced by aerobic degradation of organic-bound N, which is abundant in upper sediment (Peter et al., 1998). It was not surprising to observe that NH^+^_4_ along the depth gradient showed significant correlations with the abundance of *Alphaproteobacteria*. Taken together, bacterial community composition and diversity were driven by sediment properties, which were differed in the spatial scales (the depth and distance to the water-land junction). O_2_ is another important regulator of bacterial community structure composition and functioning. For example, O_2_ availability influences oxidation–reduction reactions in different types of wetland soils (D'angelo and Reddy, 1999). However, we failed to measure O_2_ availability because of the damage of O_2_ electrode in the field.

Overall, the sediment physiochemical characteristics had a significant effect on the diversity of beach bacterial communities along spatial gradients in the subtropical freshwater wetland at Poyang Lake. This study would improve our understanding of bacterial diversity in wetland ecosystems.

### Conflict of interest statement

The authors declare that the research was conducted in the absence of any commercial or financial relationships that could be construed as a potential conflict of interest.

## Supplementary material

Figure S1 **Relationships between the relative abundances of the dominant bacterial phyla and distance and depth**. Distance: distance to the water-land junction, depth: sediment depth to surface.

Figure S2 **Correlations between the relative abundances of the dominant bacterial phyla and the sediment physiochemical characteristics**. WC, water content; SBD, sediment bulk density; SOC, soil organic carbon.

Table S1 **All 30 soil physicochemical data**.

Table S2 **Correlation coefficients between all soil physicochemical variables**.

Table S3 **Analyses of the 30 bacterial clone libraries**.

Table S4 **Relative abundance distribution of operational taxonomic units (OTU) (97% similarity)**.

Table S5 **Correlations between the function matrix and the first two axes of the CCA microbial community ordination scores based on intraset scores**.

Table S6 **Relative abundance at phylum level (CCA)**.

Table S7 **Geochemical characteristics (CCA)**.

Table S8 **Correlations between the function matrix and the first two axes of the PCA microbial community ordination scores based on intraset scores**.
