# Supplementary figures and images for: Spatial distribution of bacterial communities driven by multiple environmental factors in a beach wetland of the largest freshwater lake in China
Source: Front Microbiol. 2015 Feb 26;6:129. doi: 10.3389/fmicb.2015.00129 (PMC4341555; doi:10.3389/fmicb.2015.00129)

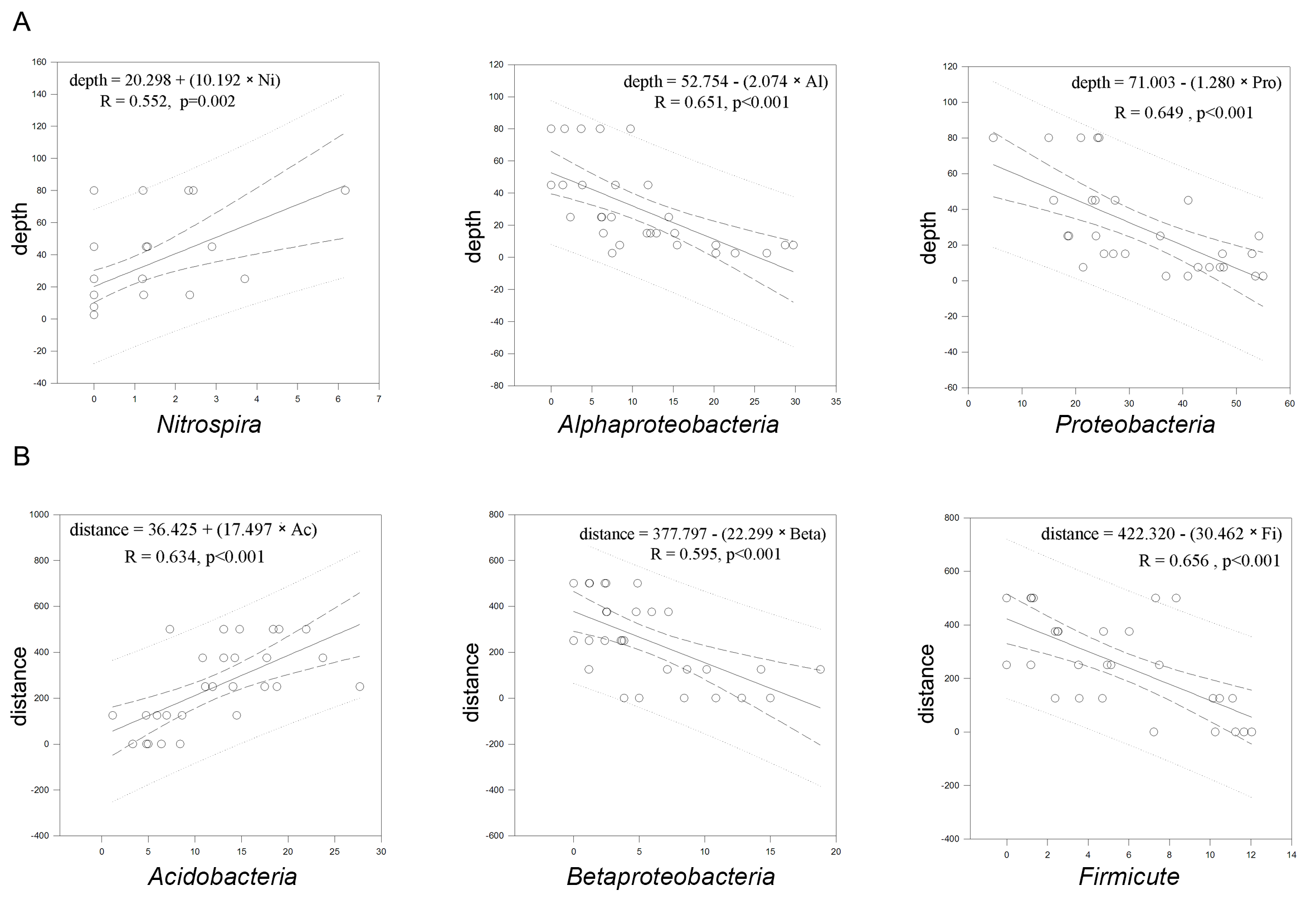

Supplement: Figure S1 — Relationships between the relative abundances of the dominant bacterial phyla and distance and depth. Distance: distance to the water-land junction, depth: sediment depth to surface. [file FigureS1.TIF]

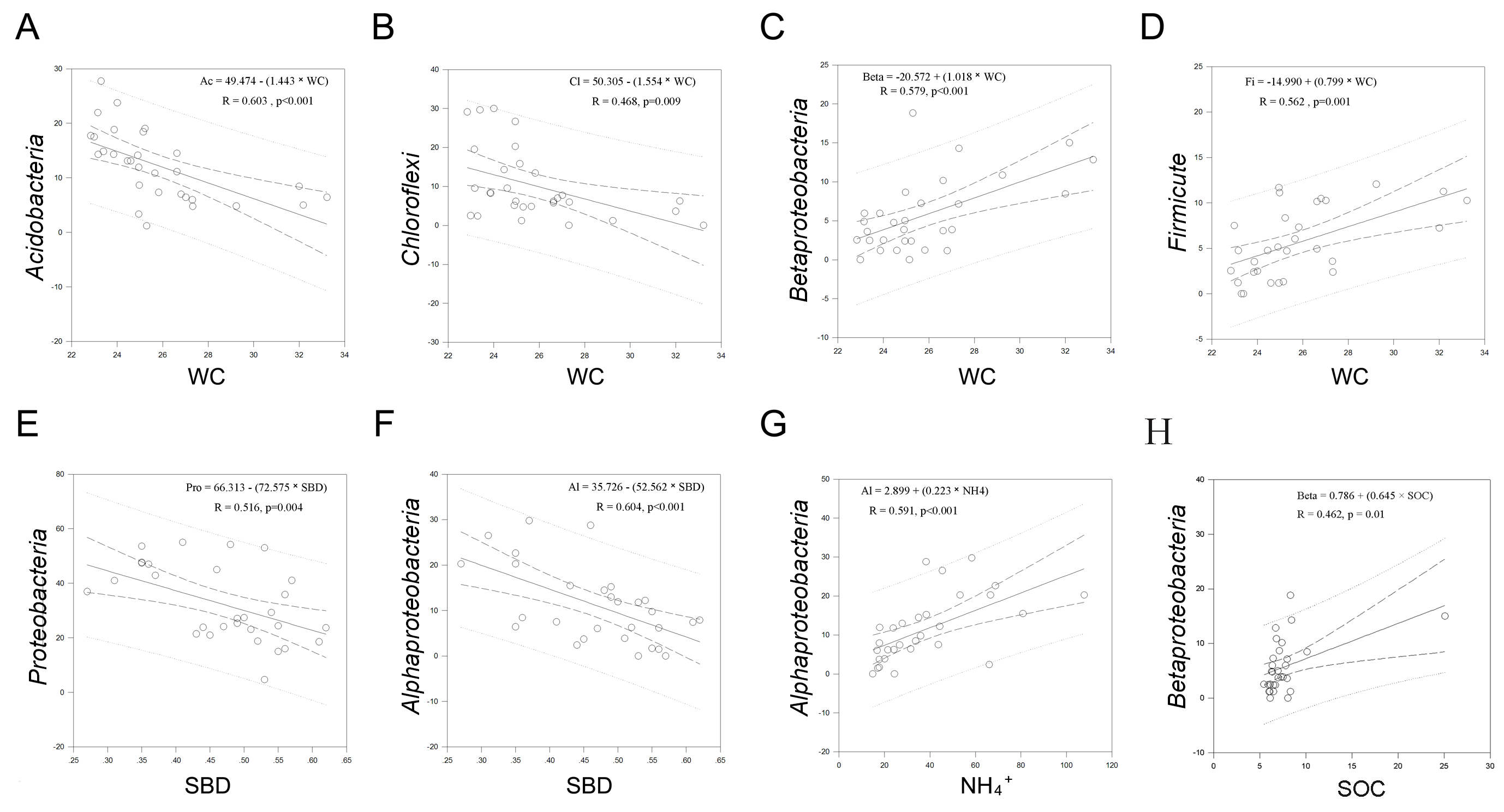

Supplement: Figure S2 — Correlations between the relative abundances of the dominant bacterial phyla and the sediment physiochemical characteristics. WC, water content; SBD, sediment bulk density; SOC, soil organic carbon. [file FigureS2.TIF]
