# Supplementary material for: Spatial distribution of bacterial communities driven by multiple environmental factors in a beach wetland of the largest freshwater lake in China
Source: Front Microbiol. 2015 Feb 26;6:129. doi: 10.3389/fmicb.2015.00129 (PMC4341555; doi:10.3389/fmicb.2015.00129)
Supplement: Table S5 — Correlations between the function matrix and the first two axes of the CCA microbial community ordination scores based on intraset scores. [file TableS5.PDF]

Table S5 Correlations between the function matrix and the first two axes of the CCA (Canonical Correspondence Analysis) microbial community ordination scores based on intraset scores

**Function Variable**	**Axis 1**	**Axis 2**
**Water contents (WC)**	0.912	0.294
**pH**	0.015	-0.315
**NH_4^+ concentration**	-0.290	0.707
**NO_3^- concentration**	0.123	0.303
**Soil organic carbon (SOC)**	0.429	0.420
**Soil bulk density (SBD)**	0.019	-0.948
