# Supplementary material for: Spatial distribution of bacterial communities driven by multiple environmental factors in a beach wetland of the largest freshwater lake in China
Source: Front Microbiol. 2015 Feb 26;6:129. doi: 10.3389/fmicb.2015.00129 (PMC4341555; doi:10.3389/fmicb.2015.00129)
Supplement: Table S8 — Correlations between the function matrix and the first two axes of the PCA microbial community ordination scores based on intraset scores. [file TableS8.DOC]

**Table S8** Correlations between the function matrix and the first two axes of the PCA (Principal component analyses) microbial community ordination scores based on intraset scores

**A)**

| **Function Variable** | **Axis 1** | **Axis 2** |
| --- | --- | --- |
| **1** | 1.276 | 0.615 |
| **2** | 0.669 | 0.658 |
| **3** | 0.913 | -0.390 |
| **4** | 0.016 | -1.363 |
| **5** | -0.842 | 0.108 |
| **6** | -2.033 | 0.372 |

**B)**

| **Function Variable** | **Axis 1** | **Axis 2** |
| --- | --- | --- |
| **A** | -1.875 | 0.763 |
| **B** | -1.061 | -1.020 |
| **C** | 0.962 | -0.480 |
| **D** | 0.933 | -0.404 |
| **E** | 1.041 | 1.141 |
